# Identification of the gut microbiota biomarkers associated with heat cycle and failure to enter oestrus in gilts

**DOI:** 10.1111/1751-7915.13695

**Published:** 2020-12-11

**Authors:** Zhong Wang, Hao Fu, Yunyan Zhou, Min Yan, Dong Chen, Ming Yang, Shijun Xiao, Congying Chen, Lusheng Huang

**Affiliations:** ^1^ State Key Laboratory of Pig Genetic Improvement and Production Technology Jiangxi Agricultural University Nanchang Jiangxi 330045 China; ^2^ Zhongkai University of Agriculture and Engineering Guangzhou Guangdong 510225 China

## Abstract

Failed puberty is one of the main reasons for eliminating gilts from production herds. This is often caused by disorders of sex hormones. An increasing number of studies have suggested that the gut microbiota may regulate sex hormones and vice versa. Whether the gut microbiota is involved in the failure of oestrus in gilts remains unknown. We used 16S rRNA gene sequencing, network‐based microbiota analysis and prediction of functional capacity from 16S rRNA gene sequences to explore the shifts in the gut microbiota throughout a heat cycle in 22 eight‐month‐old gilts. We found that a module of co‐occurrence networks composed of *Sphaerochaeta* and *Treponema*, co‐occurred with oestrus during a heat cycle. The mcode score of this module reflecting the stability and importance in the network achieved the highest value at the oestrus stage. We then identified bacterial biosignatures associated with the failure to show puberty in 163 gilts. *Prevotella*, *Treponema*, *Faecalibacterium*, *Oribacterium*, *Succinivibrio* and *Anaerovibrio* were enriched in gilts showing normal heat cycles, while Lachnospiraceae, *Ruminococcus*, *Coprococcus* and *Oscillospira* had higher abundance in gilts failing to show puberty. Prediction of functional capacity of the gut microbiome identified a lesser abundance of the pathway ‘retinol metabolism’ in gilts that failed to undergo puberty. This pathway was also significantly associated with those bacterial taxa involved in failed puberty identified in this study (*P* < 0.05). This result suggests that the changed gut bacteria might result in a disorder of retinol metabolism, and this may be an explanation for the failure to enter oestrus.

## Graphical abstract

We identified bacterial biosignatures associated with the failure to show puberty in 163 gilts. Prediction of functional capacity of the gut microbiome identified a less abundance of the pathway ‘retinol metabolism’ in gilts that failed to undergo puberty. Correlation analysis suggests that the changed gut bacteria might lead to a disorder of retinol metabolism, and further result in the failure to enter oestrus.

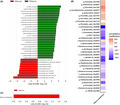

## Introduction

Puberty in gilts is defined as the first expressed oestrus with ovulation; this is a critical developmental phase indicating that the gilts can be prepared for mating and reproduction (Lents *et al*., 2020). Gilts usually reach puberty at the age of 6 months, although the timing varies within and between gilts (Palmert and Boepple, 2001, Barb *et al*., 2006, Choi and Yoo, 2013). The rate of pubertal development and successful pregnancy in gilts significantly influences the efficient management of breeding sows, and decreased age at puberty has a favourable effect on lifetime productivity of sows (Roongsitthichai *et al*., 2014). However, in the modern commercial pig industry, selection for fast growth and high lean meat percentage has resulted in a delay in the onset of puberty (Rauw *et al*., 1998). Furthermore, approximately 30% of gilts have to be removed from a production herd due to failure to enter oestrus up to the age when gilts should exhibit puberty (Saito *et al*., 2011; Lents *et al*., 2020), thereby causing significant economic loss.

The onset of puberty in gilts is regulated by the hypothalamic‐pituitary‐ovarian axis (HPOA) (Pelletier *et al*., 1981). Oestrus is initiated by the release of gonadotropin‐releasing hormone (GnRH) from the hypothalamus. The rising levels of circulating oestrogen in early puberty stages stimulate the expression of kisspeptin that further promotes GnRH release (Mayer *et al*., 2010). This feedback regulation leads to the high level of oestrogen that triggers the onset of puberty. Emerging evidence suggests that oestrogens interact with gut microbiota through the oestrogen‐gut microbiome axis (Baker *et al*., 2017; Park *et al*., 2018; Acharya *et al*., 2019; Antwis *et al*., 2019). A functional gene repertoire known as the ‘estrobolome’ in the gut microbiota has been reported to be involved in the metabolism of oestrogens (Plottel and Blaser, 2011). For example, oestrogens are metabolized by microbial‐secreted β‐glucuronidase that deconjugates glucuronide‐linked oestrogens into active forms, which alters circulating oestrogen level (Plottel and Blaser, 2011; Baker *et al*., 2017). Conversely, the diversity and composition of gut microbiota are also affected by oestrogen (Flores *et al*., 2012; Huang *et al*., 2017). For instance, the presence or absence of the oestrogen receptor in gut epithelia alters the composition of the gut microbiota of female mice (Menon *et al*., 2013). Ovariectomy can decrease the richness of the gut microbiota (Park *et al*., 2018). Low‐dose brain oestrogen and progesterone prevents menopausal syndrome by maintaining the diversity of the gut microbiome in oestrogen‐deficient rats (Park *et al*., 2018). Oestradiol profoundly altered the gut microbiota of female ob/ob mice fed a high‐fat diet (Acharya *et al*., 2019). This bi‐directional interaction between oestrogen and the gut microbiota suggests that failure to undergo oestrus in gilts may be associated with intestinal dysbacteriosis.

A recent report indicated that the relative abundances of *Aerococcaceae*, *Atopostipes*, *Carnobacteriaceae* and *Solobacterium* were associated with breeding success, hormone metabolites and ovarian cycles in the eastern black rhino (Antwis *et al*., 2019). However, to our knowledge, there has been no report concerning the correlation of gut microbiota with oestrus of gilts. The main objectives of this study were to analyse the gut microbial composition of gilts, to examine the effects of sampling batch, oestrus stage, farm and host genetics on the diversity of the gut microbiome, and especially to identify potential bacterial biosignatures associated with the gilt heat cycle and failed puberty. The analysis was performed using 16S rRNA gene sequencing, network‐based gut microbiota analysis and predicted functional capacity from the 16S rRNA sequencing data.

## Results

### The factors influencing the diversity of gut microbiota in gilts

We obtained 8,772,199 high‐quality reads from 251 samples (34,949 reads per sample) that were clustered into 6,997 operational taxonomic units (OTUs) at 97% sequence similarity. We found a total of 26 bacterial phyla that were represented in the gut microbiota of the tested gilts, nine of which had 100% prevalence. There were six phyla having an average of relative abundance > 1% in the tested samples: Firmicutes (43.9%), Bacteroidetes (38.7%), Spirochaetes (8.5%), Proteobacteria (13%), Fusobacteria (3.7%) and Tenericutes (1.6%). We then explored the effects of the host and environmental factors (sampling batch, oestrus stage, farm and host genetics) on the composition of gut microbiota via permutational multivariate analysis of variance (PERMANOVA) (McArdle and Anderson, 2001) using the Adonis function in the vegan package in R (R Core Team, 2019) by calculating the effect size (*R* ^2^) that reflected the contribution and significance of each factor to the gut microbial composition (He *et al*., 2018). The *R* ^2^ values of each factor on gut microbial variation were 0.108 (sampling batch, *P* = 0.001), 0.050 (oestrus stage, *P* = 0.034), 0.047 (farm, *P* = 0.001) and 0.037 (host genetics, *P* = 0.007). These results indicated that all four factors affected the gut microbial composition to some extent. Among these factors, the sampling batch showed the largest effect followed by oestrus stage, farm and host genetics.

**Fig. 1:**
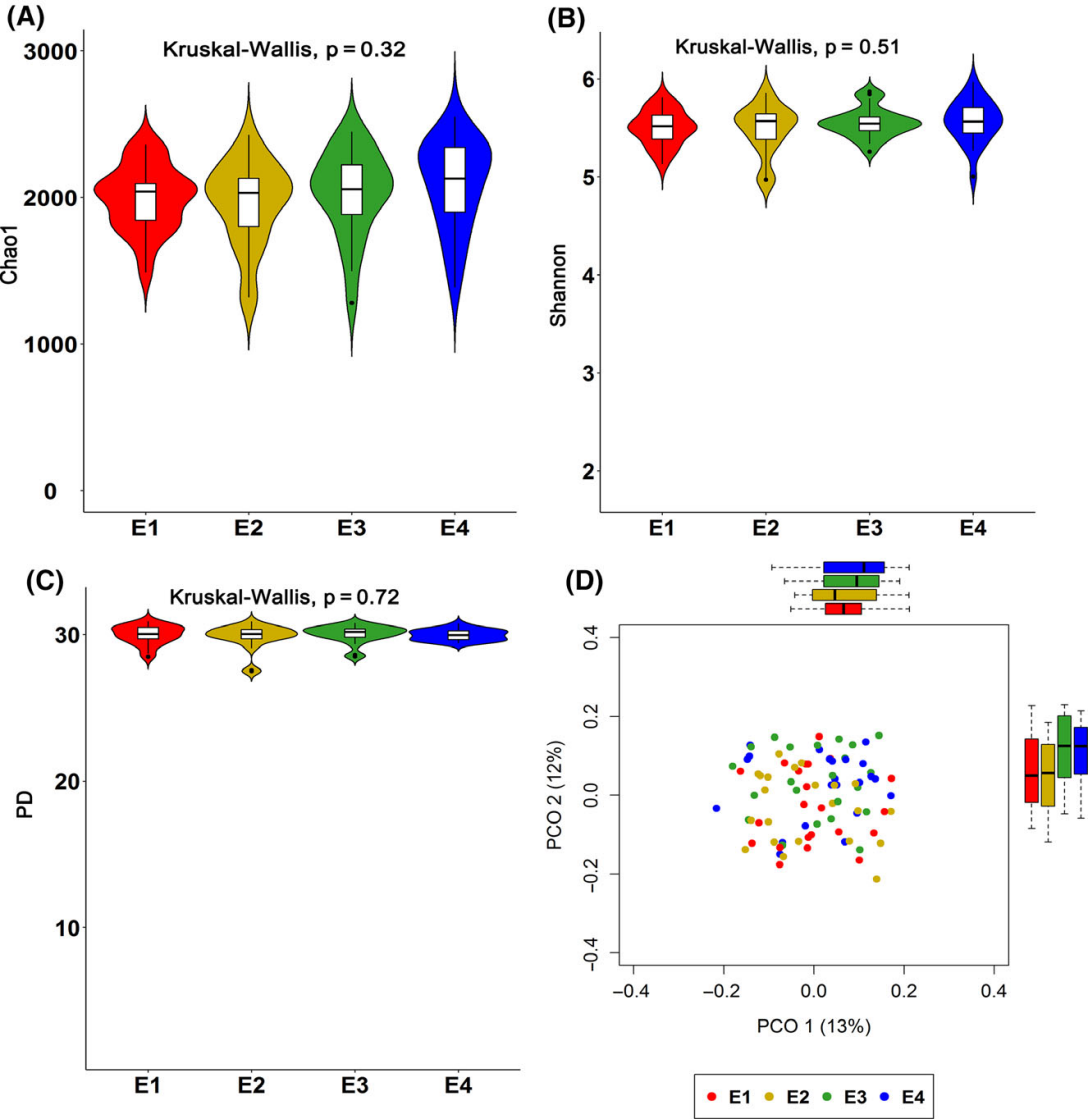
Comparison of the α‐ and β‐diversity of gut microbiota among four stages of a heat cycle (*n* = 22). Chao1 (A), Shannon (B) and phylogenetic diversity (PD) indices (C) of gut microbial communities among four stages of a heat cycle in gilts. D. Principal coordinates analysis (PCoA) of gut microbial communities based on unweighted Unifrac distance among four stages of a heat cycle in gilts (*n* = 22). Boxplots are included to visualize the differences of gut microbial compositions among four stages. E1 = Dioestrus, E2 = Pro‐oestrus, E3 = Oestrus and E4 = Metoestrus.

To further evaluate the effect of the oestrus cycle on the gut microbial composition of gilts, we first compared the α‐diversity of the gut microbiota among four stages in a heat cycle (pro‐oestrus, oestrus, metoestrus and dioestrus). Neither the Chao1 (Chao, 1984), Shannon (Shannon, 1948) or Faith’s diversity (phylogenetic diversity, PD) (Faith, 1992) indices showed significant differences among the four stages, although both Chao1 and Shannon indices were increased from dioestrus to metoestrus (Fig. 1A–C) (Kruskal–Wallis test, *P* = 0.32, 0.51 and 0.72, respectively), suggesting that the α‐diversity of the gut microbiota in gilts was not significantly affected by the heat cycle.

Principal coordinate analysis (PCoA) based on unweighted Unifrac distance (Lozupone and Knight, 2005) was performed to evaluate the changes in β‐diversity of the gut microbiota along with the heat cycle. From PCO1, significantly higher β‐diversity was observed at the pro‐oestrus stage, but no significant differences were detected among the other three stages. From PCO2, significantly higher β‐diversity was observed at the oestrus stage than at the other three stages (Fig. 1D).

### Distinct co‐occurrence networks of the gut microbiota among four stages of a heat cycle

**Fig. 2:**
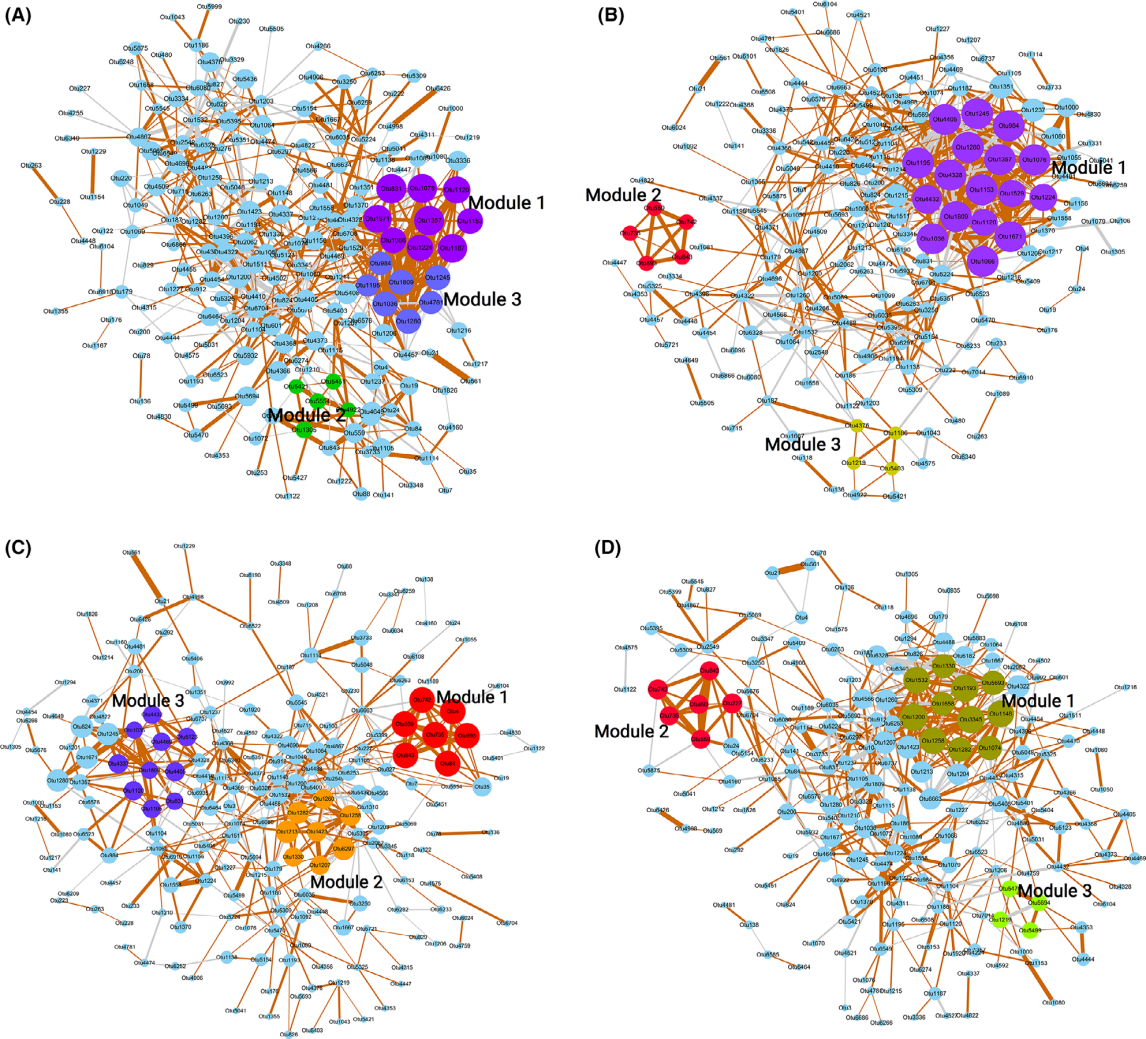
The phylogenetic co‐occurrence networks of gilt gut microbiota among four stages of a heat cycle: A. dioestrus; B. pro‐oestrus; C. oestrus; D. metoestrus. The networks were constructed at the OTU level. Node sizes are proportional to the mcode score, and different colours represent the modules within networks. The same colour indicates similar modules. The lines connecting two nodes represent SparCC correlation coefficients between the connected nodes, with the line width representing the correlation magnitude. Brown and grey lines represent significant positive and negative correlations between two OTUs, respectively, with an absolute value of correlation coefficient >0.65. Unconnected nodes were omitted.

To investigate the shifts of interaction networks of the gut microbiota at different stages of a heat cycle, we constructed co‐occurrence networks of the gut microbiota with the OTUs satisfying the following two criteria (Liu *et al*., 2019): (i) Relative abundance was > 0.05% and (ii) the OTUs were identified in more than 20% of the tested samples. Totals of 165, 145, 149 and 155 OTUs were selected for dioestrus, pro‐oestrus, oestrus and metoestrus stages respectively. The co‐occurrence networks were constructed using the SparCC algorithm (Friedman and Alm, 2012) based on OTU abundances. Clustering coefficients were calculated by the Molecular Complex Detection (MCODE) plugin in Cytoscape, and modularity was used to identify modules in the co‐occurrence networks. The stability of the network, which was reflected by the percentage of negative interactions (competition) (Coyte *et al*., 2015), obviously decreased from 31.65% at the dioestrus stage to 27.25% at the oestrus stage, and then increased to 29.33% at the metoestrus stage, indicating that the co‐occurrence networks of the gut microbiota had the highest stability at the dioestrus stage and the lowest stability at the oestrus stage. In addition, the complexity (Bader and Hogue, 2003) of the phylogenetic co‐occurrence networks, which was reflected in the average number of edges per node, was the lowest (2.97) at the stage of oestrus, and then, the complexity scores increased from metoestrus to pro‐oestrus, achieving the maximum at the stage of pro‐oestrus (4.11) (Fig. 2 and Table S1).

Modules of the co‐occurrence network of gut microbiota were further analysed for each stage. The bacteria clustered in the modules of each network are shown in detail in Table S2. Interestingly, a module containing *Sphaerochaeta* and *Treponema* (module 2 at the pro‐oestrus stage, module 1 at the oestrus stage and module 2 at the metoestrus stage) was observed since gilts entered the heat cycle. More importantly, this module had the highest mcode score. The higher the mcode score, the greater the density of nodes observed in the co‐occurrence network at the oestrus stage, suggesting that this module was more stable and more important in the networks. *Treponema* has been reported to take part in sex hormone conversion (Clark and Soory, 2006), and *Sphaerochaeta* was revealed to be involved in glucose metabolism by glycolytic and pentose phosphate pathways (Caro‐Quintero *et al*., 2012). The coexistence between this module and the oestrus cycle in gilts indicated that the two may be interrelated. In addition, the modules that were composed of OTUs annotated to *Prevotella* existed in the co‐occurrence networks of all four stages, i.e. module 1 and module 2 at the dioestrus stage, module 1 at the pro‐oestrus stage, module 3 at the oestrus stage and module 1 at the metoestrus stage, and these had the highest stability and complexity. This indicated that these modules should not be affected by the changes in sex hormones along with the stages of the heat cycle. This also implies that these modules may be involved in the basic physiological functions of metabolism in gilts.

### Identification of bacterial taxa showing distinct abundance among four stages of a heat cycle

**Fig. 3:**
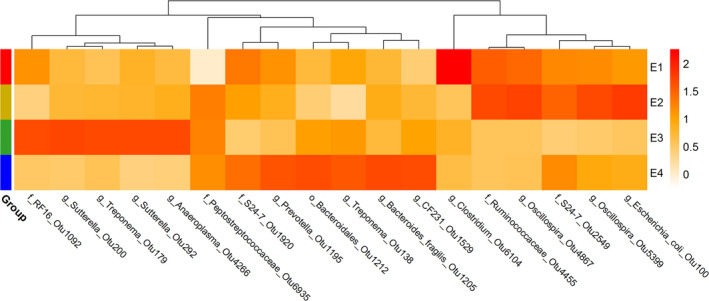
The OTUs enriched in each of four stages of a heat cycle. E1 = Dioestrus, E2 = Pro‐oestrus, E3 = Oestrus and E4 = Metoestrus.

We further identified the OTUs that were enriched in each of the four stages of a heat cycle using linear discriminant analysis with effect size estimation (LEfSe) (Segata *et al*., 2011). At the significance threshold of LDA > 2.0 and *P* < 0.05, OTU6104 (*Clostridium*) was the only bacterial taxon that was enriched at the dioestrus stage. The abundance of OTU6104 achieved the highest level in the gut at the dioestrus stage and then decreased rapidly when the gilts entered the oestrus cycle. A previous report indicated that *Clostridium scindens* can convert glucocorticoids into androgens (Ridlon *et al*., 2013). As is well known, androgen and oestrogen can be interconverted (Hammes and Levin, 2019). A rapid increase in oestrogen level after gilts entered a heat cycle may have an inhibitory effect on *Clostridium*, leading to a rapid decrease in the abundance of *Clostridium*. At the pro‐oestrus stage, *Oscillospira* (OTU4867 and OTU5399), Ruminococcaceae (OTU4455)*, Escherichia_coli* (OTU100) and S24‐7 (OTU2549) had the highest abundances in the guts of the gilts. At the oestrus stage, *Treponema* (OTU179), *Sutterella* (OTU200 and OTU292), *Anaeroplasma* (OTU4266) and RF16 (OTU1092) were enriched in the microbiota of the gilts. The abundances of these OTUs increased gradually from the dioestrus to the oestrus stage and then decreased at the metoestrus stage (the details are presented in Table S3). This change was consistent with the shift in oestrogen level during a heat cycle in gilts, suggesting that significant correlations should exist between these OTUs and oestrogen level. There were six OTUs enriched in the gut microbiota of gilts at the metoestrus stage, including *Bacteroides fragilis* (OTU1205), Bacteroidales (OTU1212), S24‐7 (OTU1920), *Treponema* (OTU138), *Prevotella* (OTU1195) and CF231(OTU1529) (Fig. 3 and Table S3). Notably, the relative abundances of *Treponema* (OTU138 and OTU179) were higher at the oestrus and metoestrus stages than at the dioestrus or pro‐oestrus stages, consistent with the results of the co‐occurrence network analysis. This further suggests that oestrogen may promote propagation of *Treponema*. These biological taxa could be used as biosignatures of the corresponding oestrus stage.

### Comparison of the gut microbial compositions between gilts showing a normal heat cycle and failure to enter oestrus

**Fig. 4:**
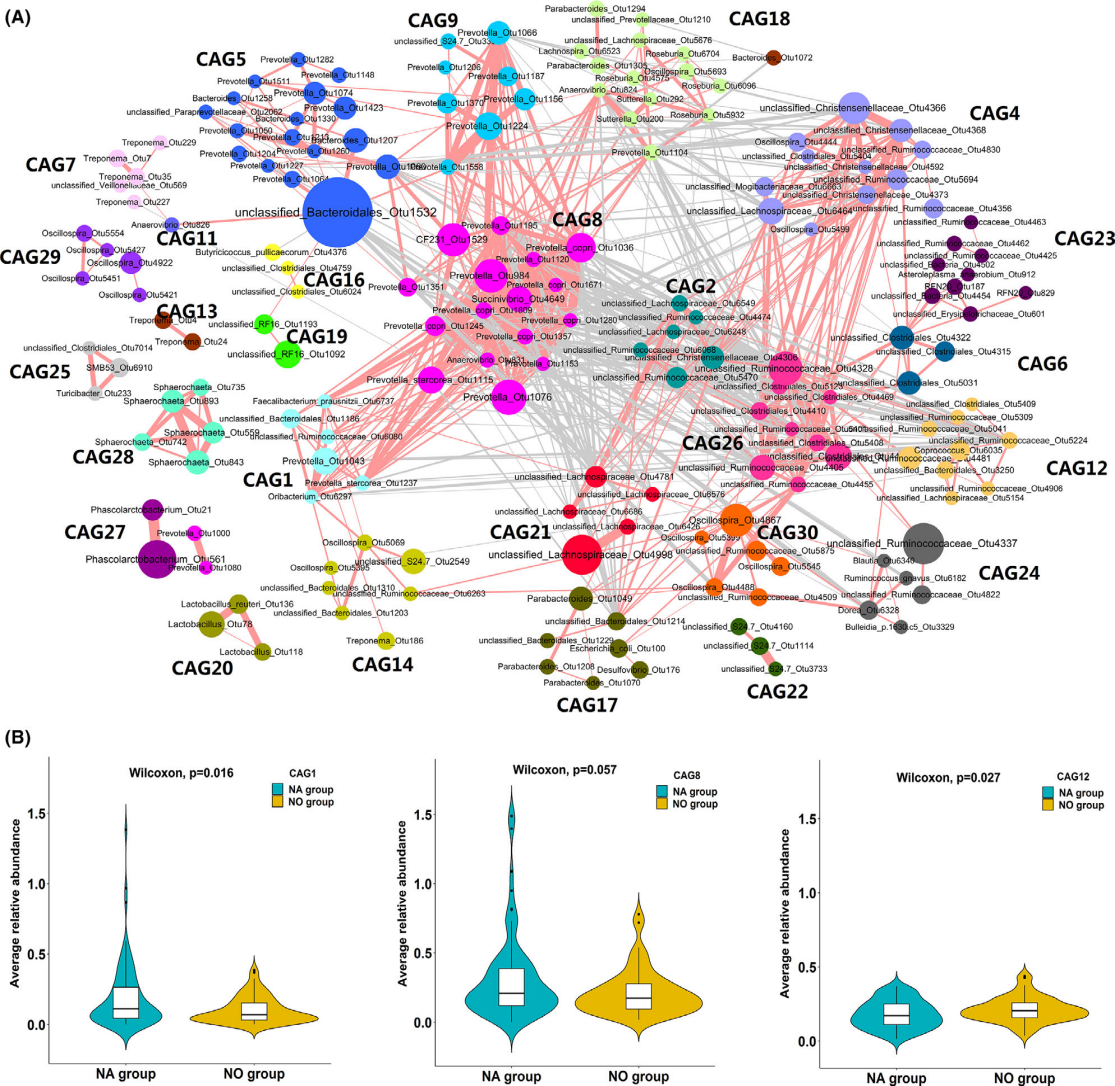
Co‐abundance groups (CAGs) of gut microbiota based on the relative abundances of OTUs and the association of CAGs with failure to enter oestrus in gilts. A. CAGs of gut microbiota based on the relative abundances of OTUs in all tested samples. A total of 302 OTUs were clustered into 30 CAGs using permutational multivariate analysis of variance. Node sizes are proportional to the average abundance of each OTU. The lines connecting two nodes represent SparCC correlation coefficients between the connected nodes, with the line width representing the correlation magnitude. Pink and grey lines represent significantly positive and negative correlations between two OTUs, respectively, with an absolute value of correlation coefficient > 0.5. Unconnected nodes were omitted. B. Association of CAGs with failure to enter oestrus in gilts. CAG1 and CAG8 were enriched in gilts having a normal heat cycle (NA group), and CAG12 had higher abundance in gilts showing failure to enter oestrus (NO group).

The gut microbiota is a complex ecosystem (Ramayo‐Caldas *et al*., 2016). Microbes in this ecosystem interact with each other and form an interaction network that affects the physiology of their host (Ke *et al*., 2019). To identify the potential functional groups composed of a variety of microbiota related to the onset of puberty in the intestinal microecosystem, we clustered OTUs into co‐abundance groups (CAGs). Those OTUs with relative abundance > 0.05% and existing in > 20% of the tested samples were used to construct CAGs using the Ward clustering algorithm based on SparCC correlation coefficients. Permutational MANOVA was used to test the statistical significance of CAG clustering using 999 permutations with Bray–Curtis dissimilarity. A total of 302 OTUs were clustered into 30 CAGs (Fig. 4A). Positive correlations were observed among CAGs 1, 5, 8, 9 and 18, and among CAGs 2, 4, 12, 21, 26 and 30. These two CAG groups were negatively associated with each other. We identified three CAGs showing significantly different abundance between gilts showing a normal heat cycle and those failing to enter oestrus: CAG1 and CAG8 showed higher abundance in gilts having a normal heat cycle (Wilcoxon rank sum test, *P* = 0.016 and 0.057, respectively), and CAG12 had higher abundance in gilts showing failure to enter oestrus (Wilcoxon rank sum test, *P* = 0.027). These CAGs should be candidate biomarkers for distinguishing normal and oestrus‐failed gilts (Fig. 4B). There were eight OTUs that were mainly annotated to *Prevotella*, *Treponema, Faecalibacterium*, *Oribacterium,* Ruminococcaceae and Bacteroidales in CAG1. It was worth noting that *Treponema* was annotated to this *o*estrus‐related CAG. Twenty OTUs were gathered in CAG8, most of which were annotated to *Prevotella* (16/20), while the other four OTUs were annotated to *Anaerovibrio*, *Succinivibrio*, CF231 and *Lachnospira*, suggesting that *Prevotella* may play an important role in ensuring oestrus in gilts. It is worth noting that the *Prevotella*‐related network was also found in the oestrus stage. As mentioned above, *Prevotella* are involved in the basic physiological functions of metabolism in gilts. CAG12, which was enriched in the gut of non‐oestrus gilts, contained 17 OTUs annotated to Bacteroidales, Clostridiales, Ruminococcaceae, Bacteroidaceae, Lachnospiraceae, *Ruminococcus*, *Coprococcus* and *Oscillospira* (Table S4). A study of PCOS (Liu *et al*., 2017a) reported that Lachnospiraceae and *Ruminococcus* were positively correlated with testosterone. This suggested that interactions between bacterial taxa and sex hormones may be a causal factor for the non‐oestrus occurrence in gilts.

### Identification of specific bacterial taxa associated with the failure to enter oestrus in gilts

**Fig. 5:**
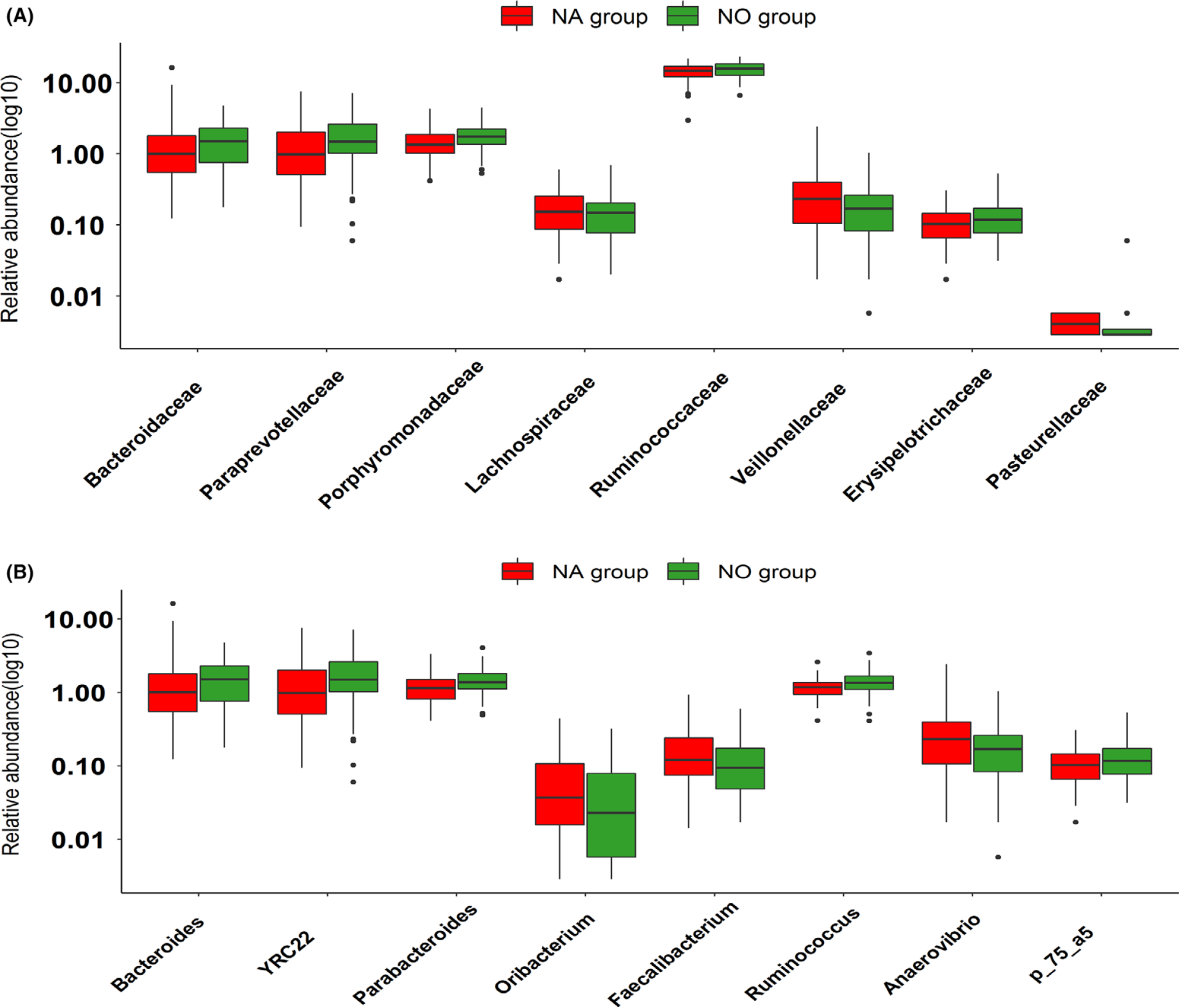
Identification of bacterial taxa by LEfSe showing differential abundances between gilts having a normal heat cycle (NA group) and gilts showing failure to enter oestrus (NO group) at the family (A) and genus (B) levels.

**Fig. 6:**
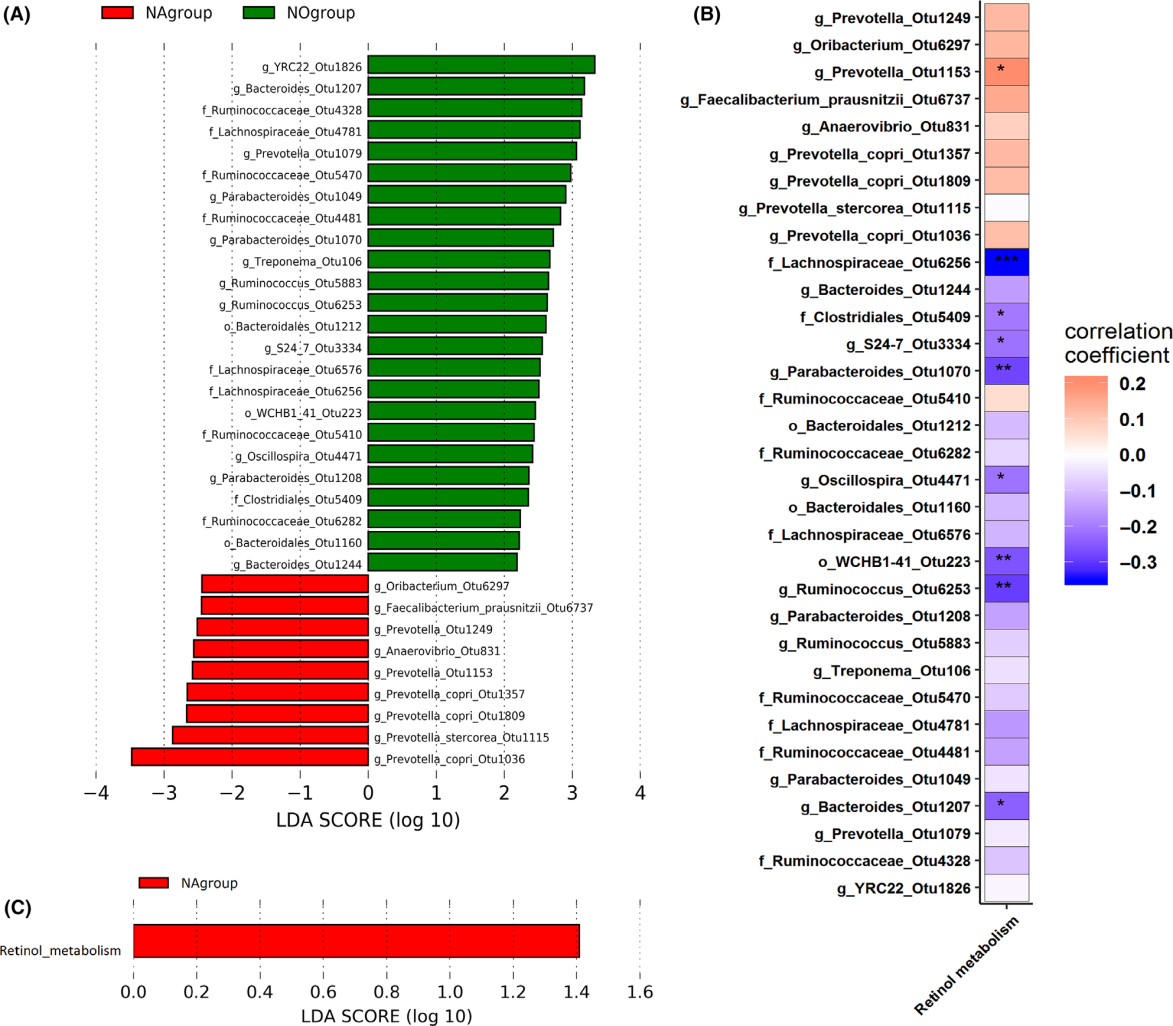
Identification of the OTUs and potential functional capacity associated with failure to enter oestrus by LEfSe. A. The OTUs associated with failure to enter oestrus in gilts by LEfSe. B. The potential functional capacity associated with failure to enter oestrus. The taxa or pathway enriched in gilts having a normal heat cycle (NA group) are indicated by a positive LDA score (green), and those taxa enriched in gilts showing failure to enter oestrus (NO group) are indicated by a negative score (red). C. The OTUs significantly associated with the pathway ‘Retinol metabolism’ (*P < 0.05; **P < 0.01 and ***P < 0.001).

We first compared the α‐ and β‐diversity of gut microbiota between gilts showing normal heat cycles and those failing to enter oestrus. The results indicated that the α‐diversity (Fig. S1A–C) and β‐diversity (Fig. S1D) of gut microbiota were not significantly different between the two groups. An LEfSe analysis was performed to identify bacterial taxa enriched in each of the two groups of gilts. At the significance threshold of LDA > 2.0 and *P* < 0.05, we detected several bacterial taxa showing different abundances between the two types of gilts. At the family level, eight bacterial taxa showed significant differences in relative abundance, including Ruminococcaceae, Bacteroidaceae, Paraprevotellaceae, Porphyromonadaceae and Erysipelotrichaceae, that were enriched in the non‐oestrus gilts. However, Lachnospiraceae, Veillonellaceae and Pasteurellaceae were significantly enriched in the gut microbiota of gilts having normal heat cycles (Fig. 5A). There were also eight genera that differed in average relative abundance between non‐oestrus gilts and gilts showing normal heat cycles. *Bacteroides*, YRC22, *Parabacteroides*, *Ruminococcus* and *p_75_a5* had greater abundance in the non‐oestrus gilts, while *Oribacterium*, *Faecalibacterium* and *Anaerovibrio* were enriched in gilts showing normal heat cycles (Fig. 6B). These results were consistent with those obtained in the CAG‐based analysis (see above), indicating that these bacterial taxa may play an important role in the failure to enter oestrus. At the OTU level, nine OTUs were enriched in gilts showing natural oestrus, and 24 OTUs were enriched in non‐oestrus gilts (Table S5). Most of the OTUs enriched in gilts showing natural oestrus were annotated to *Prevotella* (5/9), including three OTUs belonging to *Prevotella copri*. The other four OTUs were annotated to *Faecalibacterium* (two OTUs), *Anaerovibrio* and *Oribacterium*. However, the OTUs enriched in non‐oetsrus gilts were annotated to Ruminococcaceae, Lachnospiraceae and Bacteroides (Fig. 6A).

### Changes of the predicted function capacity of the gut microbiota in gilts showing failure to enter oestrus

In order to uncover whether the altered community structure of the gut microbiota changed the predicted functional capacity of the metagenome between gilts showing normal heat cycles and those failing to enter oestrus, phylogenetic investigation of communities by reconstruction of unobserved states (PICRUSt) analysis was employed to predict the Kyoto Encyclopedia of Genes and Genomes (KEGG) pathways of the gut microbiome based on 16S rRNA gene sequencing data. A total of 328 potential functional pathways were recognized and quantified. Only ‘retinol metabolism’ showed differential abundance between the two types of gilts (LDA > 1.0, *P* < 0.05), and this pathway was enriched in the gilts having a natural oestrus cycle (Fig. 6B). Correlation analysis via Spearman’s rank correlation revealed a significantly positive correlation between ‘retinol metabolism’ and *Prevotella*_OTU 1153 (*r* = 0.205, *P* = 0.033), and significantly negative correlations between ‘retinol metabolism’ and the relative abundances of *Bacteroides*_OTU1207 (*r* = −0.242, *Oscillospira*_OTU4471 (*r* = −0.212, *P* = 0.028), *Parabacteroides*_OTU1070 (*r* = −0.280, *P* = 0.003), S24‐7_OTU3334 (*r* = −0.213, *P* = 0.029) and Lachnospiraceae_OTU6256 (*r* = −0.350, *P* < 0.001) (Fig. 6C and Table S6).

## Discussion

The gut microbiota can degrade hormones and change host gene expression, thereby affecting reproductive success. In humans, circulating oestrogen can be altered by the disruption of the gut microbiome, resulting in oestrogen‐related pathologies, including infertility (Baker *et al*., 2017). However, to our knowledge, there are no reports concerning the correlation between gut microbiota and puberty onset in gilts. In this study, we evaluated the relationship between gut microbiota and the failure to enter oestrus in gilts, and we explored the compositional and potential functional characteristics of the gut microbiota at four different stages of a heat cycle. The results should further our understanding of the role of gut microbiota in puberty onset in gilts.

Consistent with previous studies, the dominant bacterial phyla in gilts were *Firmicutes, Bacteroidetes, Spirochaetes* and *Proteobacteria* (Pajarillo *et al*., 2014; He *et al*., 2019). We found a tendency of increasing α‐diversity of the gut microbiota from the dioestrus to metoestrus stages in a heat cycle, indicating that the host physiological status of oestrus can result in remodelling the microbial community composition. This result was consistent with studies of other animals (Miller *et al*., 2017; Antwis *et al*., 2019) and humans (Koren *et al*., 2012).

We examined the structure of microbial ecosystems in different stages throughout the oestrus cycle. Interestingly, a module composed of *Sphaerochaeta* and *Treponema* was found in the co‐occurrence network of the gut microbiota from the pro‐oestrus, oestrus and metoestrus stages, but not at the dioestrus stage, suggesting that this module might be involved in the heat cycle. In this module, *Sphaerochaeta* participates in glucose metabolism through glycolytic and pentose phosphate pathways (Caro‐Quintero *et al*., 2012). It is well known that gilts reduce or even lose their appetite when they are in a heat cycle (Knox, 2016). The increased abundance of *Sphaerochaeta* in the gut can enhance the host’s energy harvest, thereby compensating for the deficiency of energy caused by the reduced feed intake of gilts in heat. A previous study found a significant interaction between *Treponema* and host sex hormones (Clark and Soory, 2006). The metabolites related to androgen metabolism are negatively correlated with *Treponema* (He *et al*., 2019). *Treponema* is an important sex‐biased bacteria in humans, and steroid hormones should be the important regulators for *Treponema* (Clark and Soory, 2006). In addition, a module composed of *Prevotella* exists throughout the whole heat cycle and was not affected by oestrus. *Prevotella* is the most abundant genus in the swine gut microbiome, and it participates in acetate and succinate production resulting in acetate, formate, propionate and succinate (Oliphant and Allen‐Vercoe, 2019). Moreover, *Prevotella* is involved in synthesis of branched‐chain amino acids (BCAAs). BCAAs are essential amino acids in the human body (Pedersen *et al*., 2016). We deduced that as important gut bacteria, *Prevotella* participate in basic metabolism and hence should not be affected by the shifts in sex hormones throughout the heat cycle.

*Prevotella, Treponema, Faecalibacterium*, *Oribacterium*, *Succinivibrio* and *Anaerovibrio* were enriched in gilts showing a normal heat cycle, while Ruminococcaceae, Lachnospiraceae*, Ruminococcus, Coprococcus* and *Oscillospira* had higher abundances in gilts showing failure to enter oestrus in both the co‐abundance group and taxonomy level analyses. Similarly, a study of PCOS in women, a disease whose primary symptoms are hyperandrogenism, oligo‐ or amenorrhoea and polycystic ovaries (Fauser *et al*., 2012), suggested that the abundances of Ruminococca, Lachnospiraceae and *Oscillospira* in the gut were significantly different between women with the disease and normal controls. These bacteria are associated with the regulation of sex hormones (Moreno‐Indias *et al*., 2016; Liu *et al*. 2017a; Torres *et al*., 2018; Thackray, 2019). As mentioned above, *Treponema* is also an important regulator of steroid hormones (Clark and Soory, 2006). *Faecalibacterium* participates in the butyryl CoA: acetyl CoA transferase pathway resulting in butyrate, carbon dioxide and hydrogen formate production (Oliphant and Allen‐Vercoe, 2019). *Anaerovibrio* was negatively associated with porcine fatness (He *et al*., 2016), and *Anaerovibrio lipolytica* can produce lipase in hydrolysis of triglycerides (Henderson, 1971). *Succinivibrio* are important bacteria producing succinate (Pope *et al*., 2011). It is well known that steroids can directly promote oestrus in sows. However, energy (Zhou *et al*., 2014) and lipids (Tummaruk *et al*., 2009) are indirectly related to oestrus initiation in gilts. The above bacteria might jointly promote oestrus onset through regulating sex hormones and host energy metabolism.

We identified the KEGG pathway ‘retinol metabolism’ that was enriched in gilts showing a normal heat cycle. Retinol (vitamin A) plays an important role in the regulation of animal reproduction. For example, retinol participates in ovarian steroidogenesis and oocyte maturation (Brown *et al*., 2003), in meiosis during foetal ovarian development and postnatal testis development (Spiller *et al*., 2012), and also in protecting oocytes against heat stress during maturation (Maya‐Soriano *et al*., 2013). Retinol deficiencies in cycling animals may cause reductions in ovarian size and steroid production, eventually leading to reproductive senescence (Juneja *et al*., 1966; Ganguly *et al*., 1971). The experimental gilts used in this study were raised on farms located in the subtropical region, and the faecal samples were collected from May to October, when the temperature ranged from 28°C to 35°C. Heat stress is one of the known causes leading to failure to enter oestrus in gilts (Zhao *et al*., 2015). Heat stress significantly affects the nuclear and cytoplasmic maturation of oocytes (Andreu‐Vazquez *et al*., 2010). Retinol metabolism could prevent oocyte apoptosis caused by heat stress, thereby promoting ovarian steroidogenesis and oocyte maturation and in turn facilitating oestrus cycle onset in gilts. Retinol metabolism has been related to gut microbiota such as *Bifidobacterium* (Liu *et al*., 2017b) and *Akkermansia* (Huda *et al*., 2019). In this study, the OTUs that were annotated to *Bifidobacterium* and *Akkermansia* did not reach significance level in LEfSe analyses between normal oestrus and non‐oestrus gilts. This could be due to there being other bacteria with unknown functions that also participate in retinol metabolism in the guts of gilts. However, we found that *Prevotella* was enriched in gilts showing natural oestrus. Correlation analysis revealed that *Prevotella* was positively related to ‘retinol metabolism’, implying that *Prevotella* may be involved in retinol metabolism and thereby affect oestrus onset. In addition, several bacterial taxa enriched in non‐oestrus gilts, such as Lachnospiraceae, *Ruminococcus* and *Oscillospira,* had significant negative correlations with ‘retinol metabolism’ suggesting that these bacteria taxa might lead to non‐oestrus in gilts by inhibiting retinol metabolism. Therefore, disorders of retinol metabolism resulting from dysbacteriosis may be another important factor in the failure to enter oestrus.

As for the limitations of this study, we did not measure the concentration of blood sex hormones (e.g., oestrogen) or metabolites related to retinol metabolism in tested samples, because it was difficult to harvest blood samples from gilts with a certain sample size. Therefore, we cannot confirm the possible mechanisms of gut microbiota affecting gilt oestrus. Furthermore, metagenomic sequencing should be performed in future studies to investigate bacterial species and their functional capacities associated with failure to enter oestrus; this will provide the candidate species for isolation and the causality confirmation in germ‐free animals.

## Experimental procedures

### Animals, phenotyping and sample collection

Two experimental gilt cohorts were used in this study. One cohort was raised on Yangjiang farm and comprised Yorkshire and Landrace × Yorkshire gilts, and the other cohort was housed in Lianzhou farm and included Landrace × Yorkshire gilts. Gilts were quarantined before entering the breeding farm, where they were examined for porcine reproductive and respiratory syndrome virus, pseudorabies virus and classical swine fever virus. Only those pigs showing negative for all above disease markers were included in the study. Heat observation was preliminarily performed twice a day from the age of 6 months using the method of behaviour observation for sow oestrus (‘standing’ to boars and back pressure). A heat cycle of 21 days was divided into four stages of pro‐oestrus, oestrus, metoestrus and dioestrus according to the definitions described by Senger (2003). We defined the day when the sow started showing ‘standing’ to boars (about 1.5 days: day 0 to day 1.5, vulval swelling) as day 0. In the heat period (oestrus stage), blood oestrogen in gilts achieves the highest level, and ovulation occurs at the end of this period. The pro‐oestrus stage (from day −3 to 0) is characterized by visible behavioural symptoms such as vulva reddening and mounting behaviour. Metoestrus (from day 1.5 to 5) is the stage when the vulva subsides and no standing behaviour occurs; in this stage, follicle cells are transformed into luteal cells and form a corpus luteum. The dioestrus stage (from day 5 to 18) is marked by a fully functional corpus luteum and a high progesterone concentration (Fig. S2). Because it is difficult to harvest blood samples from gilts at multiple time points to determine the peak of the level of blood oestrogen or oestradiol, it was a limitation of the study that we did not measure the level of blood oestrogen. If the gilts had not displayed oestrus behaviour up to 9.5 months of age (more than five heat cycles could be observed in normal gilts), they were defined as failure to enter oestrus gilts (non‐oestrus group). The full‐siblings or half‐siblings of non‐oestrus gilts displaying natural oestrus cycles (NA group) were selected as the control cohort. We collected 251 faecal samples from 185 gilts in three batches, among which 163 faecal samples were harvested from gilts showing failure to enter oestrus (73 samples) and their counterparts having a normal heat cycle (90 samples) at the dioestrus stage at the age of 7 months. The samples were used to test the association between the composition of gut microbiota and the phenotype of failure to enter oestrus. To investigate the impact of different stages of the heat cycle on the composition of gut microbiota, the other 88 faecal samples were collected from 22 gilts at four different stages of the heat cycle around the age of 8 months (22 samples for each of pro‐oestrus, oestrus, metoestrus and dioestrus). All gilts were provided the same formula diet four times daily, and water was available ad libitum from nipple dispensers. None of the gilts had been given any antibiotics within 1 month before faecal sample collection. Faeces were collected from the anus of gilts in the morning (between 8:00 and 10:00) from May to October 2018. All faecal samples were immersed in liquid nitrogen immediately after collection and then stored at −80°C until use.

### Faecal DNA extraction, 16S rRNA gene sequencing and data processing

QIAamp Fast DNA Stool Mini Kits (Qiagen, Germany) were used to extract DNA from faecal samples according to the manufacturer’s protocol. DNA concentration and purity were determined by a Nanodrop‐1000 and electrophoresis in 0.8% agarose gels. The fusion primers 338F (5′‐ACTCCTACGGGAGGCAGCAG‐3′) and 806R (5′‐GGACTACHVGGGTWTCTAAT‐3′) were used to amplify the V3–V4 region of the 16S RNA gene under an annealing temperature of 55°C with 27 cycles. The PCR amplicons were purified with agarose gels using an AxyPrep DNA Gel Extraction Kit (Corning, NewYork, USA). Sequencing was performed on an Illumina MiSeq platform (Illumina, San Diego, California, USA). All sequencing data were submitted to the China National GeneBank database (CNGBdb) (https://db.cngb.org/cnsa/) with accession number CNP0000888. Quality control of the raw data was performed to filter the primers, low‐quality reads, high nucleotide ambiguities and barcode sequences using custom scripts. The paired‐end clean reads were assembled into tags via FLASH (v.1.2.11) (Magoc and Salzberg, 2011). In order to avoid statistical bias resulting from an uneven sequencing depth, the sequence depth of each sample was rarefied to 34,949 tags. USEARCH software (v7.0.1090) and the UPARSE‐OTU algorithm were employed to cluster tags into operational taxonomic units (OTU) at 97% sequence similarity (Majaneva *et al*., 2015). The relative abundances of OTUs were calculated with Mothur (version 1.31.2) (Schloss *et al*., 2009). The RDP classifier program (v2.2) (Wang *et al*., 2007) was used to classify the representative sequences. The representative sequences for each OTU were screened for further annotation using Greengenes (DeSantis *et al*., 2006) as the reference database.

### Statistical analyses

#### Calculation and comparison of the α‐ and β‐diversity of gut microbiota

Those OTUs that had relative abundance < 0.01% and were present in < 10% of the experimental samples were removed from further analysis. Mothur (version 1.31.2) (Schloss *et al*., 2009) was used to calculate the α‐diversity of gut microbiota via the Chao1, Shannon and phylogenetic diversity (PD) indices (Shannon, 1948; Chao, 1984; Faith, 1992). Unweighted Unifrac distances were calculated to compare the β‐diversity of the gut microbial community among the four stages of a heat cycle, and between gilts showing a normal heat cycle and those failing to enter oestrus using principal coordinated analysis (PCoA) (Lozupone and Knight, 2005) by QIIME (version 1.9.1) (Caporaso et al., 2010).

#### Analysing the effects of host and environmental factors on gut microbiota

PERMANOVA was used to explore the effects of host and environmental factors including sampling batch, oestrus stage, farm and host genetics on the composition of gut microbiota. The effect size of each factor (*R* ^2^), which was used to determine the contribution and significance of all factors on gut microbial composition, was also calculated by the PERMANOVA, and the *P* values were generated based on 999 permutations (Liu *et al*., 2019).

#### Construction of co‐occurrence networks

OTUs satisfying the following two criteria were chosen to construct clustering co‐occurrence networks: (i) Relative abundance > 0.05%, and (ii) the OTUs were identified in more than 20% of the tested samples. Co‐occurrence networks of gut microbiota at dioestrus, pro‐oestrus, oestrus and metoestrus stages were inferred separately based on the SparCC algorithm (Friedman and Alm, 2012). The correlations between OTUs were calculated by the PCIT algorithm (Reverter and Chan, 2008). The confidence of the interactions between OTUs (nodes) was built with > 0.65 absolute sparse correlation coefficients. Co‐occurrence networks were visualized using Cytoscape (version 3.7.1) (Lopes *et al*., 2010). Topological characteristics of networks, including clustering coefficients, densities and scale‐free properties, were calculated by Cytoscape (Smoot *et al*., 2011). The Molecular Complex Detection (MCODE) plugin (Bader and Hogue, 2003) in Cytoscape was used to select sub‐modules based on vertices weighted by local neighbourhood density in co‐occurrence networks of gut microbiota. MCODE was also used to assess the importance of sub‐modules and to calculate the topological characteristics of main modules (i.e., node number, edge number and module importance score).

#### Construction of co‐abundance groups (CAG)

The OTUs were selected to construct the CAGs according to the criteria described in the section on construction of co‐occurrence networks. CAGs were constructed by SPIEC‐EASI in the R package. First, the interactions between OTUs were determined by computing correlation matrices based on their abundances using the SparCC algorithm. The pairwise OTUs with correlation coefficients greater than 0.5 were left for constructing CAGs. The correlation coefficient values were converted to a correlation distance (1‐correlation coefficient value), and the Ward clustering algorithm was employed to group the OTUs into each CAG using the ‘hclust’ function in the R package. Permutational MANOVA was used to test the statistical significance of each CAG clustering using 999 permutations with Bray–Curtis dissimilarity. When *P* < 0.005, the CAG was deemed acceptable.

### Identification of bacterial taxa and predicted functional capacities associated with failure to enter oestrus

To gain insight into the potential functional capacity of the gut microbiome, PICRUSt analysis was performed to predict KEGG pathways based on 16S rRNA gene sequences (Langille *et al*., 2013). The false discovery rate (FDR < 0.05) was used to determine the threshold significant *P*‐value. LEfSe analysis was performed online (http://huttenhower.sph.harvard.edu/galaxy) to identify bacterial taxa or predicted KEGG pathways that differed significantly between gilts showing natural heat cycle and the failure to enter oestrus.

## Conflict of Interest

None declared.

## Author contributions

L.H.: conceived and designed the experiments and revised the manuscript; C.C.: designed the experiments, analysed the data and wrote and revised the manuscript. Z.W.: performed the experiments, analysed the data and wrote the manuscript. H.F. and Y.Z.: analysed the data and performed the experiments. M.Y., D.C., M.Y. and S.X.: collected the samples. All authors read and approved the final manuscript.

## Ethical Approval

All animal works were conducted according to the guidelines for the care and use of experimental animals established by the Ministry of Agriculture of China. Animal Care and Use Committee in Jiangxi Agricultural University specially approved this project.

## Supporting information


**Fig S1**. Comparison of the α‐ and β‐diversity of gut microbiota between gilts showing failure to enter estrus (NO group) (n = 73) and gilts having a normal heat cycle (NA group) (n = 90). Chao1 (A), Shannon (B) and phylogenetic diversity (PD) indices (C) of gut microbial communities were compared between NO and NA gilts. D. PCoA based on unweight UniFirc distances for gut microbial composition between NO and NA gilts.

**Fig S2**. Division of a heat cycle in gilts and the times for sampling. The division of a at cycle.
