# Supplementary figures and images for: Identification of the gut microbiota biomarkers associated with heat cycle and failure to enter oestrus in gilts
Source: Microb Biotechnol. 2020 Dec 11;14(4):1316–30. doi: 10.1111/1751-7915.13695 (PMC8313273; doi:10.1111/1751-7915.13695)

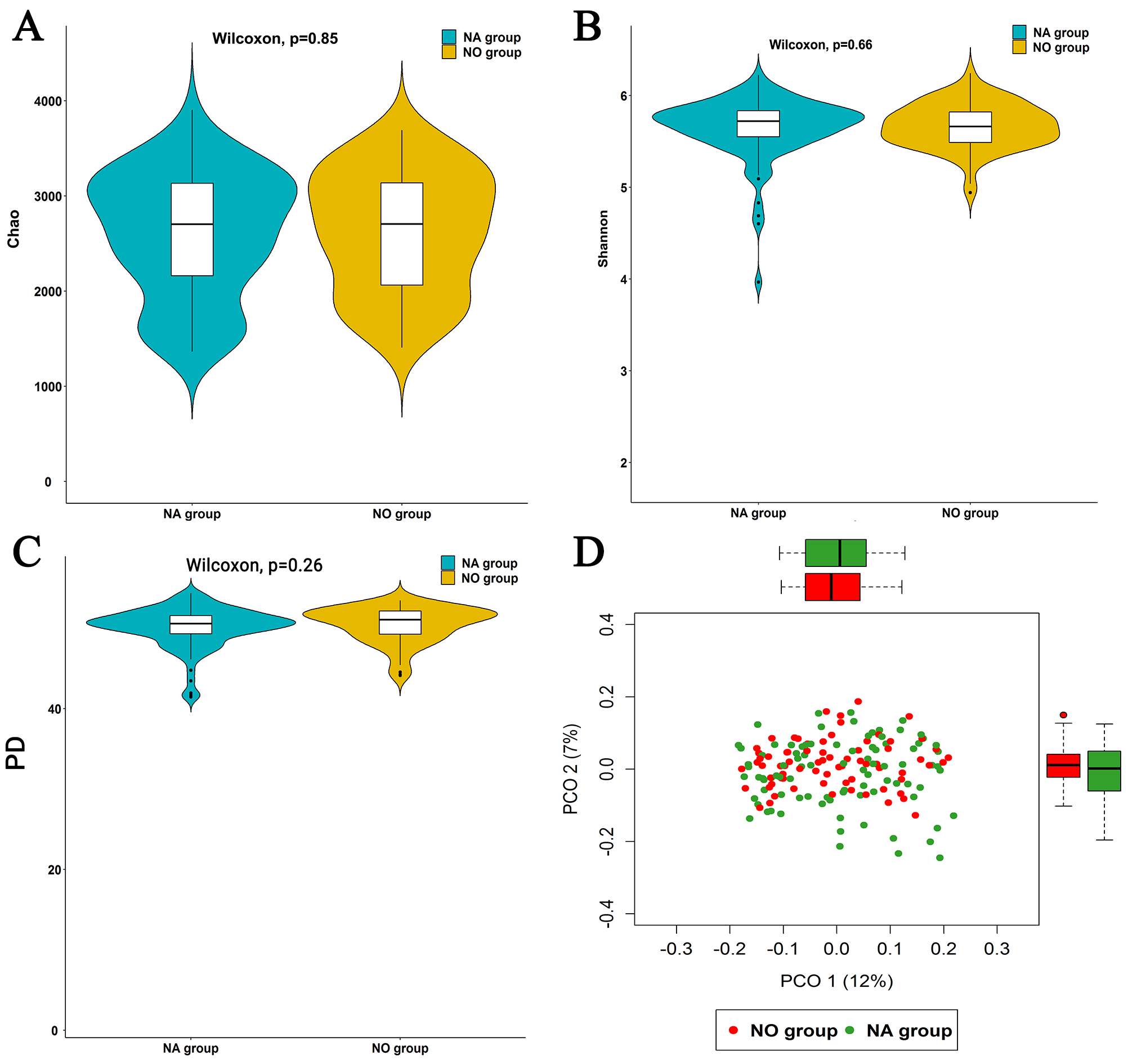

Supplement: Supplementary file 1 — Fig S1. Comparison of the α‐ and β‐diversity of gut microbiota between gilts showing failure to enter estrus (NO group) (n = 73) and gilts having a normal heat cycle (NA group) (n = 90). Chao1 (A), Shannon (B) and phylogenetic diversity (PD) indices (C) of gut microbial communities were compared between NO and NA gilts. D. PCoA based on unweight UniFirc distances for gut microbial composition between NO and NA gilts. [file MBT2-14-1316-s001.tif]

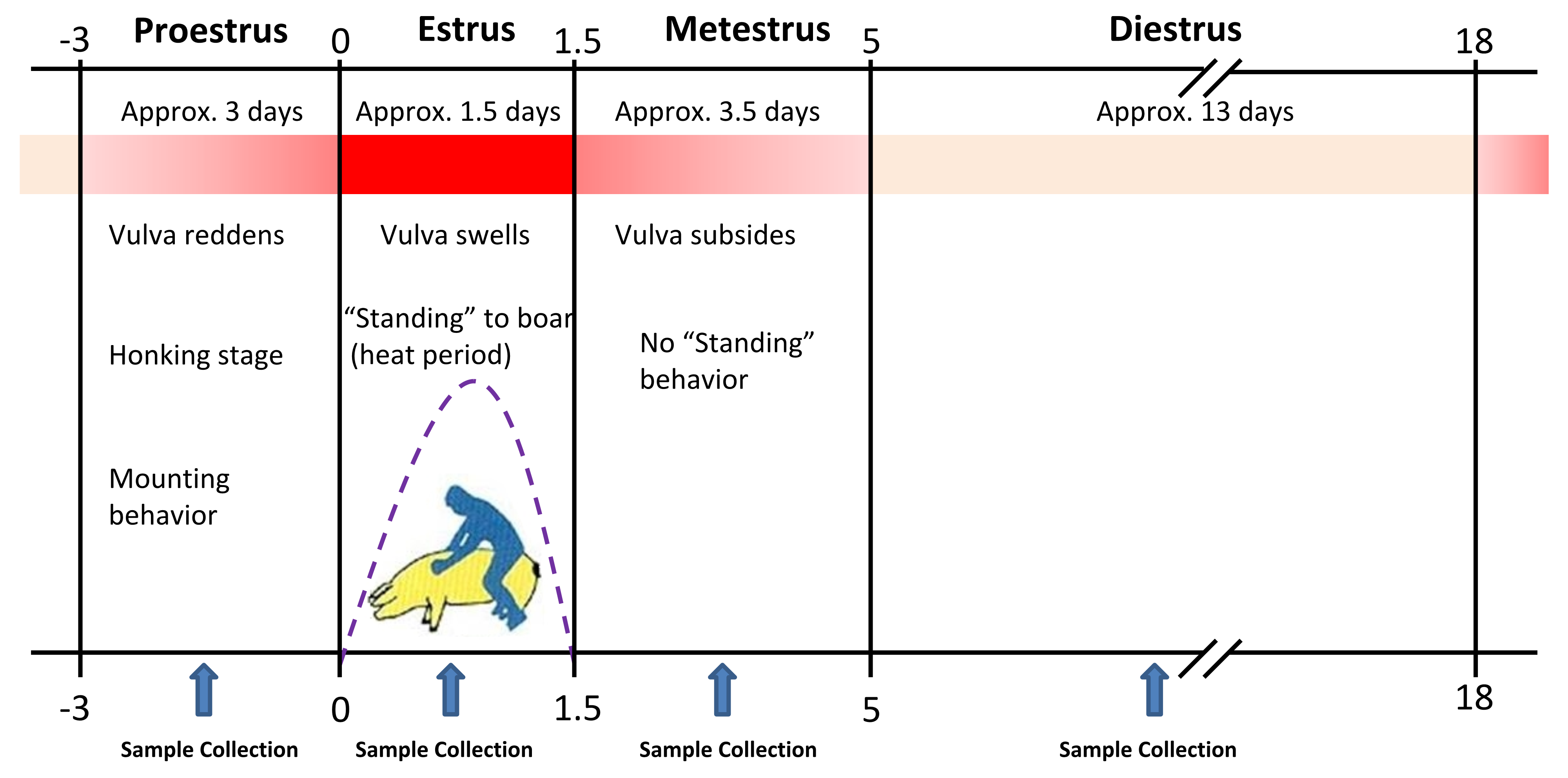

Supplement: Supplementary file 2 — Fig S2. Division of a heat cycle in gilts and the times for sampling. The division of a at cycle. [file MBT2-14-1316-s002.tif]
